# Emotional intelligence impact on academic achievement and psychological well-being among university students: the mediating role of positive psychological characteristics

**DOI:** 10.1186/s40359-024-01886-4

**Published:** 2024-07-12

**Authors:** Ye Shengyao, Lin Xuefen, Hashem Salarzadeh Jenatabadi, Nadia Samsudin, Ke Chunchun, Zahari Ishak

**Affiliations:** 1 Department of Public Education, Wenzhou Vocational College of Science and Technology, Wenzhou, Zhejiang Province China; 2 Faculty of Social Sciences and Liberal Arts, UCSI University, Kuala Lumpur, Malaysia; 3 Department of Science and Technology Studies, Faculty of Science, Universiti Malaya, Kuala Lumpur, Malaysia

**Keywords:** Emotional intelligence, Psychological well-being, Positive psychological characteristics, Emotional intelligence theory and academic achievement

## Abstract

The main objective of this study is to examine the relationship of emotional intelligence with psychological well-being and academic achievement through positive psychological characteristics among university students in China. The study was conducted with postgraduate and undergraduate students. The integration of emotional intelligence theory and positive psychological theory was used in this study. The introduced framework included emotional intelligence as the main independent variable, self-efficacy, motivation, and resilience as three mediators, and psychological well-being and academic achievement as two dependent variables. A survey was conducted among 518 students, and structural equation modelling was used to analyse the data. The study found that emotional intelligence was positively related to positive psychological characteristics, psychological well-being, and academic achievement, and the effects were stronger among postgraduate students. Also, positive psychological characteristics, which include self-efficacy, motivation, and resilience, mediate the relationship between emotional intelligence and psychological well-being and academic achievement, and the relationship was stronger among postgraduate students. Proper coping strategies and mechanisms can be helpful to improve both psychological well-being and academic achievement at the same time among university students.

## Introduction

Emotional intelligence, often known as EI, refers to the capacity to recognise, control, and regulate emotions, which facilitates flexible thinking and comprehension of the significance and outcomes of emotions [1]. Since the 1990s, researchers have extensively studied emotional intelligence by investigating the potential correlations between emotional intelligence and psychological or educational results [2]. Based on the principles of educational psychology and learning, these skills should be positively associated with academic success, serving as a crucial tool to prevent academic failure [3]. Furthermore, according to Maalouf, Hallit [4] and Shafait, Khan [5], emotional intelligence serves as a significant indicator of different types wellbeing, health, and overall quality of life. Emotional intelligence is often seen as essential in both personal and professional contexts. It has an impact on our ability to regulate behaviour, negotiate intricate social situations, and make individual choices that lead to favourable outcomes. Compared to cognitive intelligence, which remains generally stable throughout the course of one’s life, emotional intelligence has the potential to be enhanced and improved with time [6].

### Emotional intelligence, academic achievement, and psychological well-being

There is a high correlation between emotional intelligence and academic success, as emotions have a significant impact on human [7]. Based on the principles of learning and teaching psychology, evolutionary development, and the school of positive psychology, there is a significant association between emotional intelligence and mental processes, focus, and self-control in stressful situations [2].

Nevertheless, there is no consensus of opinion. An argument has been made by some academics that emotional intelligence plays a moderating function in personality variables that support more efficient approaches to school activities [8]. Some studies argue that emotional intelligence has a moderating effect on academic achievement, but it is not the most significant predictor of it [9, 10]. Ultimately, more research indicates that the correlation is either absent or lacks statistical significance [11]. Hence, there is a lack of agreement among scholars regarding the role of emotional intelligence in regulating or predicting academic achievement.

A study conducted by Shuo, Xuyang [12] centred on Chinese postgraduates developed a chain mediation model utilising ecosystem theory. The study investigated the function of social support and psychological resilience as chain mediators between emotional intelligence and well-being. The study revealed a strong and positive correlation between emotional intelligence and well-being. Postgraduates with a high level of emotional intelligence shown superior abilities in seeing and understanding both their own feelings and those of others. This enhanced their communication skills and facilitated pleasant emotional encounters, ultimately contributing to a heightened sense of well-being in their daily lives. In contrast, individuals with lower emotional intelligence had a higher frequency of negative emotions and a diminished sense of overall well-being. Additionally, the study found that social support acted as a mediator between emotional intelligence and well-being. It demonstrated that emotional intelligence was associated with a greater perception of social support, which subsequently led to an enhancement in the well-being of postgraduates.

A separate empirical investigation conducted by Loi and Pryce [13] examined the correlation between emotional intelligence, student well-being, mindful self-care, and academic burnout in university students. The study demonstrated that emotional intelligence has a favourable impact on well-being and mental self-care, while being adversely correlated with several aspects of academic burnout. Significantly, the study discovered that mindful self-care acts as a mediator between emotional intelligence and specific components of academic burnout, namely tiredness and effectiveness. Nevertheless, it did not act as a mediator in the connection between emotional intelligence and cynicism. These findings indicate that cultivating emotional intelligence and mindfulness-based stress reduction in students can enhance their overall well-being and serve as a safeguard against the negative consequences of academic burnout.

These findings are consistent with the prevailing agreement in psychological research that emotional intelligence plays a crucial role in individual and group adaptation, hence enhancing mental well-being and academic achievement. Proficiently comprehending and controlling emotions are crucial aptitudes for successfully navigating the intricacies of social relationships and personal obstacles, both within academic environments and beyond.

### Mediating roles of positive psychological characteristics

Positive psychological factors/ elements/ or behaviours are characteristics that make a person happy and improve their general mental health [14]. These things are very important in the field of positive psychology, which is a branch of psychology that works on making people healthier and more effective at what they do instead of just treating mental illness [15]. These factors include optimism, resilience, gratitude, hope, self-efficacy, mindfulness, empathy, motivation, curiosity, etc. When it comes to emotional intelligence and performing well in educational institutions, self-efficacy, motivation, and resilience are the most important traits [16].

The scientific literature generally supports a moderate correlation between emotional intelligence and academic performance. However, this correlation can be influenced by other positive psychological factors such as self-efficacy [17, 18], locus of control [19, 20], self-esteem [21, 22], resilience [23, 24], and motivation [25, 26]. These factors contribute to more adaptive behaviour. A study by Baños, Calleja-Núñez [27] indicated that certain components of emotional intelligence, specifically emotional clarity and healing, had a favourable and direct impact on one’s belief in their ability to succeed academically. In addition, the ability to restore emotional well-being was found to be a significant predictor of both behavioural and emotional involvement.

#### Role of self-efficacy

Significantly, academic self-efficacy was found to be a highly effective mediator between emotional clarity and repair, and the many aspects of academic engagement. This mediation significantly enhanced behavioural and emotional involvement while reducing behavioural and emotional detachment. Students with high emotional clarity can better comprehend their emotional responses to academic problems, allowing them to retain a positive attitude and persevere in their efforts. Similarly, those with excellent emotional repair skills can recover more rapidly from setbacks and stress, allowing them to stay motivated and engaged in academic activities.

Chang and Tsai [28] constructed a model to examine the correlation between emotional intelligence, learning motivation, self-efficacy, and academic accomplishment in university students. The objective of the study was to investigate the impact of emotional intelligence on learning motivation, self-efficacy, and academic accomplishment, particularly in the setting of online courses. The study also examined the role of self-efficacy as a mediator between learning motivation and academic accomplishment, as well as whether emotional intelligence indirectly influences academic achievement through its impact on learning motivation and self-efficacy.

The primary objective of a study by Tang and He [29] was to examine how self-efficacy acts as a mediator in the connection between emotional intelligence and motivation for learning. It emphasised that self-efficacy, which refers to an individual’s overall assessment and assurance in their capacity to act, can impact cognitive growth and inspire individuals to engage in constructive behaviours. Another study by Adeyemo and Adeleye [30] revealed that self-efficacy can operate as a mediator between emotional intelligence and learning motivation. This implies that a higher level of emotional intelligence can enhance self-efficacy, which subsequently has a favourable influence on learning motivation among college students.

This study stresses the importance of self-efficacy in understanding the relationship between emotional intelligence, psychological well-being, and academic achievement. Emotional intelligence, which includes skills like emotional clarity and repair, enables people to better understand and control their emotions [31]. This, in turn, increases their self-efficacy, or belief in their ability to complete specific tasks or obstacles. High self-efficacy improves psychological well-being by instilling a sense of control and decreasing anxiety, stress, and other unpleasant emotions. When kids believe in their skills, they are more likely to engage fully with their academic work, persevere in the face of adversity, and perform better academically.

#### Role of motivation

Within the realm of higher education, academic motivation plays a pivotal role in shaping students’ educational achievements and general state of being. In academic settings, motivation is commonly characterized as the internal force that compels individuals to participate in educational activities, persist in the face of difficulties, and attain their educational objectives [32]. This inherent drive not only guides behavior towards certain objectives but also maintains the commitment and drive devoted to these pursuits over a period of time. University students’ motivation can be impacted by several aspects, such as their personal inclination towards the subject matter, the perceived significance of academic achievement, and the support and acknowledgment they receive from the educational setting.

Emotional intelligence is crucial in influencing academic motivation [33]. Individuals that possess a high level of emotional intelligence have a greater ability to comprehend and control their own emotions, as well as recognize and react to the emotions of others. This capacity enables individuals to sustain an optimistic perspective, efficiently manage stress, and adjust to evolving circumstances and obstacles. For instance, Emotional intelligence can assist a student in identifying emotions of dissatisfaction and employing effective techniques to sustain motivation when encountering challenging situations or academic obstacles [24]. Moreover, students that possess emotional intelligence tend to have heightened empathy and proficiency in their communication, so fostering improved relationships with both peers and educators [34]. Consequently, this leads to enhanced motivation through heightened social support and active involvement within the academic community.

This study proposes that motivation plays a vital role in the connection between emotional intelligence and psychological well-being and academic accomplishment. It is hypothesized that motivation acts as a mediator in this relationship. In essence, a high level of emotional intelligence promotes increased motivation, which subsequently results in enhanced psychological well-being and academic achievement. The concept of the mediating role of motivation implies that by improving emotional intelligence, student well-being and academic performance can be enhanced indirectly through the initial elevation of their motivation levels. This idea highlights the significance of cultivating emotional intelligence abilities in educational environments, not only to promote emotional and social functioning, but also to boost academic results by increasing motivation.

#### Role of resilience

Academic resilience pertains to a student’s capacity to successfully overcome difficulties, pressure, and hardships in educational environments while preserving or swiftly restoring their academic achievement [35]. It has a crucial role in the achievement and persistence of university students, as it not only affects their capacity to handle academic requirements but also effects their overall educational experience. Resilient pupils possess the ability to persist in the face of obstacles, adjust to evolving situations, and derive knowledge from their mistakes [36]. Resilience is now widely acknowledged as a crucial quality that helps students succeed in their academic pursuits, especially in the ever-changing and frequently demanding setting of higher education.

Academic resilience is significantly influenced by emotional intelligence [37]. Individuals that possess elevated levels of emotional intelligence demonstrate superior abilities in perceiving, comprehending, and controlling their own emotions as well as the emotions of others [38]. Emotional awareness and control are crucial for effectively managing stress and developing problem-solving skills, both of which are vital aspects of resilience [39]. For example, a student with high emotional intelligence may utilize their comprehension of emotional dynamics to sustain motivation and concentration when confronted with academic obstacles, or to actively pursue supportive connections that enhance their ability to bounce back from adversity. Furthermore, the self-regulatory component of emotional intelligence empowers pupils to uphold a favourable mindset and outlook, helping them to recover swiftly from failures and losses.

The correlation between resilience and psychological well-being is extensively documented [40]. Students who possess resilience are more inclined to sustain a consistent psychological state, so safeguarding themselves against mental health conditions like anxiety and depression [41]. Resilience in academic settings empowers students to effectively handle the stress that comes with demanding academic tasks and social pressures, therefore promoting their general mental health and well-being [42]. Psychological stability is essential for both personal satisfaction and academic success, since it plays a vital role in maintaining engagement and tenacity in one’s studies [43].

This study proposes that resilience plays a mediating role in the relationship between emotional intelligence and psychological well-being and academic accomplishment. The study acknowledges the substantial effects of emotional intelligence on resilience, as well as the benefits of resilience on both psychological well-being and academic achievement. This theory posits that enhancing students’ emotional intelligence could indirectly bolster their well-being and academic achievement by initially fortifying their resilience [44]. By cultivating emotional intelligence, educational programs have the potential to enhance the ability of students to bounce back from challenges, leading to improved academic performance and psychological well-being [41]. This highlights the crucial role of resilience in the success of university students.

### Comparison study between undergraduate and postgraduate students

The correlation between educational level and emotional intelligence, as well as their interplay with academic achievement, can be comprehended and represented through diverse viewpoints. As individuals advance in their schooling, they are likely to come across a variety of social circumstances and intricate challenges that can promote the growth of emotional intelligence. Higher education provides students with opportunity to participate in activities that foster self-awareness, empathy, and interpersonal skills. Studies indicate a positive correlation between elevated levels of emotional intelligence and improved academic achievement in different level of education [34, 45, 46]. The reason for this is that emotional intelligence qualities such as self-regulation, empathy, and social skills can improve a student’s capacity to negotiate the academic setting, resulting in improved results [47]. Several studies have assessed the influence of educational level on research pertaining to emotional intelligence and academic achievement [48, 49]. Nevertheless, a comparative analysis of the framework between postgraduate and undergraduate students has not been conducted previously.

### Present study

In this study we utilize Positive Psychology Theory and Emotional Intelligence Theory to investigate the influence of emotional intelligence on academic achievement and psychological well-being.

#### Positive psychology Theory

Positive psychology is a discipline that examines the factors and mechanisms that lead to the success or optimal performance of individuals, groups, and organizations, proposes that qualities like optimism, resilience, and gratitude have positive effects on academic and personal growth [50]. The study examines how positive psychological qualities, such as resilience, motivation, and self-efficacy, mediate the relationship between emotional intelligence and outcomes such as psychological well-being and academic achievement. This research builds upon previous studies in the field of human behavior [51, 52]. Studies on human behavior frequently explore the interplay between individual variances, psychological characteristics, and contextual elements in shaping diverse life consequences. This inquiry is consistent with a wide range of psychological research that highlights the significance of individual characteristics in influencing behavior and achievement in several areas of life.

#### Emotional Intelligence Theory

Emotional Intelligence Theory is a prominent idea in psychology that has found widespread application in diverse disciplines such as education, corporate behavior, and leadership. Psychologists John Mayer and Peter Salovey [53] popularized the notion in the early 1990s, and it was later expanded upon by journalist and author Daniel Goleman [54]. Within the scope of our study, Mayer and Salovey’s idea of emotional intelligence is particularly relevant. This theory offers a systematic method for evaluating and comprehending the emotional abilities that can impact the mental health and academic success of college students. By utilizing this model, the study can assess the manner in which pupils observe, employ, comprehend, and regulate their emotions, and how these capabilities are interconnected with their academic and psychological results. An example of this is the importance of efficiently managing emotions, which is likely essential for coping with academic stress and interpersonal connections in a university setting. This, in turn, can impact students’ general well-being and academic achievement.

These theories provide a strong foundation for studying how emotional intelligence, influenced by positive psychological traits, affects the academic performance and psychological well-being of college students. This comprehensive approach not only emphasizes the immediate advantages of emotional intelligence but also illuminates the fundamental psychological mechanisms that enable these advantages.

In light of this, the current study seeks to examine the following:

1. The relationship of emotional intelligence with positive psychological characteristics include (a) self-efficacy, (b) motivation and (c) resilience among undergraduate and postgraduate students.
2. The relationship of self-efficacy with (a) academic achievement and (b) psychological well-being among undergraduate and postgraduate students.
3. The relationship of motivation with (a) academic achievement and (b) psychological well-being among undergraduate and postgraduate students.
4. The relationship of resilience with (a) academic achievement and (b) psychological well-being among undergraduate and postgraduate students.
5. The mediating roles of self-efficacy, motivation and resilience between emotional intelligence and academic achievement.
6. The mediating roles of self-efficacy, motivation and resilience between emotional intelligence and psychological well-being.

**Fig. 1:**
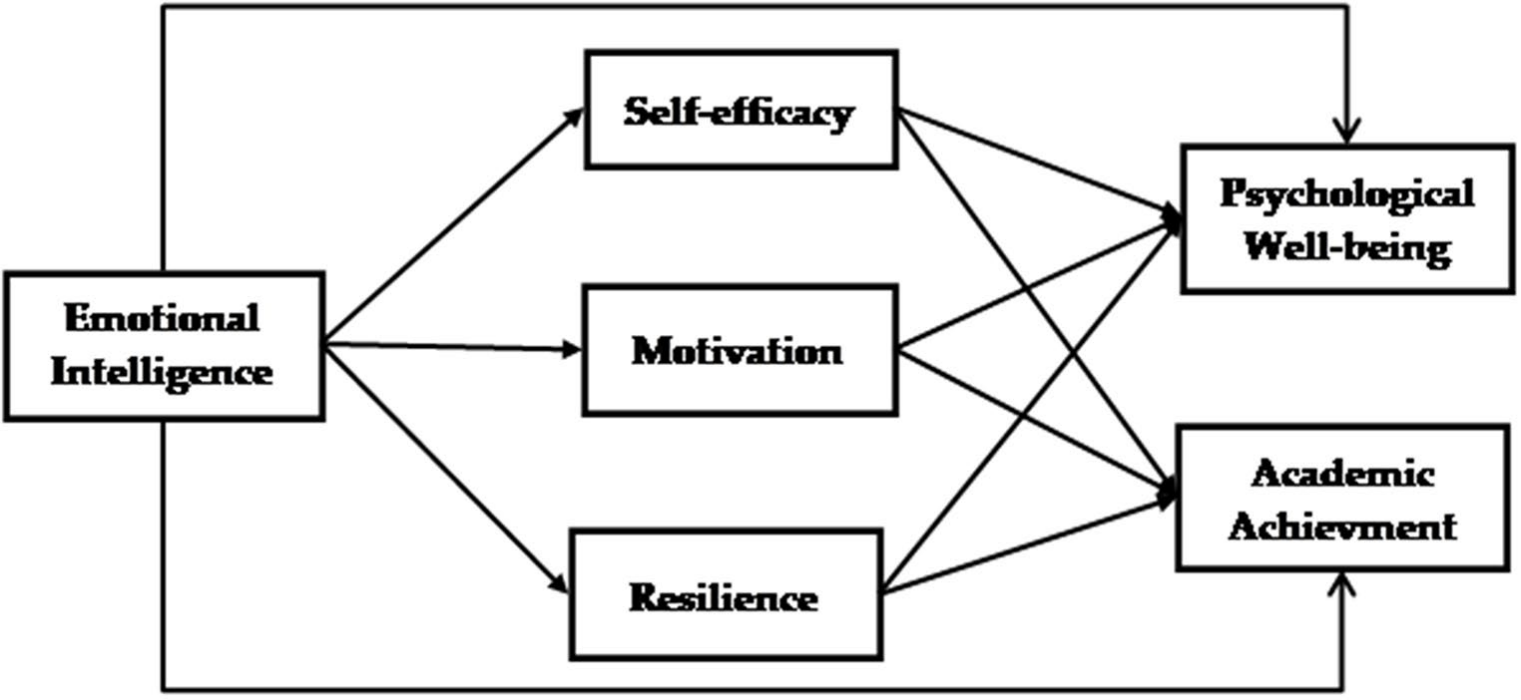
Research proposed model

Figure 1 illustrates the proposed model.

The study aims to investigate the interrelationships between emotional intelligence, motivation, resilience, psychological well-being, and academic accomplishment in university students. The purpose of these hypotheses is to define the specific routes via which emotional intelligence might influence significant outcomes for university students. The focus is on understanding how motivation and resilience can act as crucial mechanisms that mediate these effects. Every hypothesis is formulated to enhance our comprehension of the intricate relationship between individual attributes and academic achievements in higher education environments. The hypotheses are explicitly stated as follows:

##### Hypothesis 1

Emotional intelligence has a positive impact on the academic performance of university students.

##### Hypothesis 2

Emotional intelligence has a good impact on the psychological well-being of university students.

##### Hypothesis 3

Motivation acts as a mediator between emotional intelligence and academic accomplishment.

##### Hypothesis 4

Motivation acts as a mediator in the connection between emotional intelligence and psychological well-being.

##### Hypothesis 5

Resilience acts as a mediator in the connection between emotional intelligence and academic accomplishment.

##### Hypothesis 6

Resilience acts as a mediator in the connection between emotional intelligence and psychological well-being.

##### Hypothesis 7

Self-efficacy acts as a mediator in the connection between emotional intelligence and academic accomplishment.

##### Hypothesis 8

Self-efficacy acts as a mediator in the connection between emotional intelligence and psychological well-being.

### Contributions of this study

This study significantly contributes to the current research on emotional intelligence, academic resilience, and student well-being. It does so by analysing the mediating roles of motivation and resilience in these interactions in a unique manner. The following are the distinctively novel elements of this study in comparison to previous research:

Initially, mediating the role of self-efficacy, motivation, and resilience: This study diverges from past research by examining the mediating role of motivation and resilience in the links between emotional intelligence and outcomes such as academic achievement and psychological well-being. The study proposes that motivation and resilience play a crucial role in explaining how emotional intelligence affects academic and well-being results, providing a fresh viewpoint on the underlying mechanisms. This method offers a more comprehensive understanding of how emotional intelligence can be effectively utilized in educational environments, particularly in relation to process-oriented features.

Furthermore, this study incorporates three main theoretical frameworks, namely Positive Psychology Theory and Emotional Intelligence Theory, to offer a complete perspective on the interplay of emotional intelligence, academic motivation, and resilience. By amalgamating these theories, the research not only expands the comprehension of how emotional and motivational factors intertwine but also emphasizes the intricate interplay between individual aptitudes (such as emotional intelligence) and their motivational and behavioral consequences in academic settings.

The study primarily targets university students, acknowledging their crucial stage of emotional and intellectual growth. This specific group of individuals frequently experiences substantial academic and social stressors, which makes the examination of emotional intelligence, resilience, and motivation very pertinent. By customizing the research for this specific demographic, the study enhances our focused comprehension of how these concepts might be nurtured during higher education, a critical stage for individual and career growth.

Furthermore, this has practical implications for educational policies and interventions: The study provides practical implications for educational policy and interventions by showing that motivation and resilience might moderate the impact of emotional intelligence on well-being and academic success. It implies that educational programs aimed at improving emotional intelligence could be created with the dual purpose of directly influencing student outcomes and promoting resilience and motivation as intermediate objectives. By utilizing this approach, educational institutions can successfully construct focused interventions that foster these attributes, ultimately leading to improved student achievement.

## Methodology

### Research procedures

The study began by creating questionnaires that were based on past research, with each question being grounded in known theoretical frameworks and preceding empirical evidence. The study assessed the variables of emotional intelligence, academic motivation, resilience, academic accomplishment, and psychological well-being using scales that were selected based on their relevance and previous validation in similar research settings. The content and face validity of each scale were assessed by a panel of specialists to ensure that they were suitable for the intended group. Subsequently, pilot tests were performed to assess the clarity, pertinence, and general effectiveness of the questionnaires by involving a limited sample of the intended population. The pilot’s feedback was utilized to enhance the questionnaires, specifically targeting any uncertainties or misunderstandings encountered by the participants. The sampling procedures were defined to guarantee a representative sample of university students, employing stratified random sampling to consider characteristics such as age, gender, and academic discipline. The surveys’ original validity and reliability were then reevaluated using the utilization of exploratory factor analysis (EFA).

### Measurement scales

The current study utilised measurement scales that have previously undergone rigorous testing and validation by other researchers. The students’ responses were evaluated using a 5-point Likert scale ranging from 1 (representing significant disagreement) to 5 (representing strong agreement). Table 1 presents a thorough overview of the theoretical literature and research questions pertaining to latent variables.

#### Emotional intelligence

The Wong and Law Emotional Intelligence Scale or WLEIS [55] was used to assess emotional intelligence. This scale consisted of 16 items. The questionnaire consists of four subscales, each including four items: self-emotion appraisals (SEA), others’ emotion appraisals (OEA), regulation of emotion (ROE), and use of emotion (UOE). SEA, this element of emotional intelligence entails knowing one’s emotional states, how they affect ideas and behaviours, and the intricacies and complexity of personal emotional responses [56]. High self-emotion evaluation indicates a thorough understanding of one’s emotional environment, which is essential for emotional management and relationships. The OEA measure a person’s ability to read others’ emotions. Effective communication and interpersonal skills require this competence. It entails reading others’ feelings, empathising with them, and interpreting them socially. High OEA competency helps people grasp others’ emotions and viewpoints, improving relationship management, conflict resolution, and social understanding [57]. ROE refers to a person’s emotional control. This dimension entails learning to control emotions, especially in difficult situations, and use emotional knowledge to attain goals and adapt to different contexts [58]. Psychological well-being and healthy personal and professional interactions depend on emotion management. High ROE shows adaptive and productive emotion management [59]. UOE is the ability to use emotions to improve cognition and performance. This dimension measures a person’s ability to use emotional awareness for good in problem-solving and decision-making [60]. High UOE scores indicate that a person can integrate emotional information into their cognitive process, using their emotional knowledge to direct their actions and improve task performance [61].

#### Emotional intelligence

Self-efficacy: Self-efficacy is measured based on Rowbotham and Schmitz [62] based on 10 questions. Sample questions included (1) “*I know that I can maintain a positive attitude toward this course even when tensions arise*”, (2) “*I am confident in my ability to learn*,* even if I am having a bad day.*”, and (3) “*I know that I can finish the assigned projects and earn the grade I want*,* even when others think I can’t*.”

#### Motivation

Harter [63] employed a 33-item motivation measure with two subscales, Intrinsic and Extrinsic. The Intrinsic Motivation Subscale includes curiosity, challenge, and independent mastery; the Extrinsic Motivation Subscale includes pleasing teacher, dependence on teacher, and easy work.

1. Intrinsic Motivation Subscale (17 items).

A. curiosity (3 items).
B. challenge (9 items).
C. independent mastery (5 items).

2. Extrinsic Motivation Subscale (16 items).

A. pleasing teacher (4 items).
B. dependence on the teacher (6 items).
C. easy work (6 items).

The minimum and maximum scores of this scale are 33 and 165, respectively. Values ranging from 33 to 66 indicate a low degree of academic motivation, values between 66 and 99 suggest an average level of academic motivation, and values above 99 indicate a high level of academic drive [64].

#### Resilience

Cassidy [65] introduced a motivation assessment consisting of 30 items, divided into three distinct subscales containing (a) Negative affect and emotional response (7 items), (b) Reflecting and adaptive help-seeking (9 items), and (c) Perseverance (14 items).

Psychological Well-being: Ryff’s Scales of Psychological Well-being (SPWB) is used to measure psychological well-being [66]. The SPWB comprises a total of 42 elements distributed among six distinct dimensions, including (1) Autonomy, (2) Environmental Mastery, (3) Personal Growth, (4) Positive Relations, (5) Purpose in Life, and (6) Self-acceptance.

#### Academic Achievement

The assessment of academic achievement was conducted by the self-reported cumulative grade point average (GPA) obtained during college. To facilitate further analysis, the GPA variable was transformed into a categorical variable. This study employed four primary GPA categories: “Below 2.50”, “2.51–3.00”, “3.01–3.50”, and “3.51–4.00”. These categories were respectively designated as bad, average, good, and exceptional.

**Table 1:** Theoretical support for measurement variables

Latent Variable	Quantity of inquiries	Theoretical support	
Emotional Intelligence	16 items	Wong and Law [55]	
Self-efficacy	10 items	Rowbotham and Schmitz [62]	
Motivation	33 items	Harter [63]	
Resilience	30 items	Cassidy [65]	
Psychological Well-being	42 items	Ryff and Singer [66]	

### Data analysis strategies

The study employed a systematic approach to data analysis, commencing with descriptive statistics to present an outline of the data’s distribution, central tendencies, and variability for each variable, namely emotional intelligence, academic motivation, resilience, academic achievement, and psychological well-being. After doing the initial analysis, we used Structural Equation Modelling (SEM) with AMOS software to evaluate the expected connections between variables. The analysis was divided into multiple crucial subsections: The study initially assessed the robustness of the measurement model by examining its validity, reliability, and multicollinearity. The constructs’ validity and internal consistency were evaluated using factor loadings, Cronbach’s alpha, and the Variance Inflation Factor (VIF) respectively, to ensure their accuracy and absence of collinearity. Model fitting entails assessing the adequacy of the model’s fit to the data by utilizing measures such as the Chi-Square goodness-of-fit statistic, Root Mean Square Error of Approximation (RMSEA), Comparative Fit Index (CFI), and Tucker-Lewis Index (TLI). This process ensures that the model accurately reflects the observed data. Common Method Variance (CMV) was mitigated by implementing measures to account for potential bias resulting from the data collecting approach. This was commonly done by doing Harman’s single-factor test or employing other statistical techniques to identify any artifact variance. The Structural Model study involved investigating the direct and indirect impacts among the components, revealing the mediating functions of motivation and resilience in the relationship between emotional intelligence and results in academic performance and psychological well-being. The use of an all-encompassing strategy in SEM facilitated a thorough comprehension of the fundamental mechanisms and the robustness of the connections within the suggested model.

The study utilized a multigroup analysis to compare the structural links between undergraduate and postgraduate student groups, in addition to the comprehensive SEM analysis. This analysis was crucial in identifying whether the relationships established in the structural model, such as the impact of emotional intelligence on academic achievement and psychological well-being through the mediation of motivation and resilience, varied across different educational levels. The multigroup SEM approach entailed executing the identical model individually for each group and thereafter contrasting the path coefficients to ascertain whether there were statistically significant disparities between undergraduates and postgraduates. The completion of this phase was crucial in comprehending the potential impact of the educational environment on the dynamics of emotional intelligence and its subsequent outcomes. By conducting this study, researchers could gain specific and customized knowledge that could be used to create focused interventions for various educational levels. This would improve the success of programs designed to enhance emotional intelligence, motivation, resilience, and academic performance for undergraduate and postgraduate students, taking into account their individual needs.

### Pilot study and sampling process

A pilot study was conducted by delivering a total of 100 surveys to business students at different educational institutions in the provinces of Zhejiang, Shanghai, and Jiangsu, China. There were 83 valid responses, yielding a response rate of 83%. The G*Power software was utilised to do a power analysis, which showed that a minimum sample size of 512 individuals is required for the inquiry. The computation was conducted using an expected effect size of 0.2, a predetermined alpha value of 0.05, and an estimated power of 0.85. The questionnaires were originally prepared in English and then subjected to a comprehensive evaluation by two experts who possess expertise in both Chinese and English. This procedure entailed utilising a translation and subsequent retranslation technique to guarantee precision. The initial results from the pilot survey indicate that the measuring constructs shown satisfactory levels of reliability and validity. The utilization of internal consistency and construct validity played a crucial role in guaranteeing the reliability and validity of the results in this investigation.

#### Internal consistency

Cronbach’s alpha was used to evaluate the internal consistency of the scales used to measure emotional intelligence (0.761), self-efficacy (0.802), motivation (0.702), resilience (0.765), psychological well-being (0.813), and academic accomplishment (0.776). A Cronbach’s alpha value above 0.70 shows strong internal consistency among the items on a scale, implying that the responses were dependable across different items of the same test.

#### Construct validity

In order to establish the construct validity, the study employed both confirmatory factor analysis (CFA) and exploratory factor analysis (EFA). An exploratory factor analysis (EFA) was initially undertaken to investigate the underlying structure of the scales and validate the factor structure proposed in the original scale. Subsequently, Confirmatory Factor Analysis (CFA) was employed to validate the factor structure that was revealed during Exploratory Factor Analysis (EFA), so confirming that the scales accurately assessed the required constructs. The Kaiser-Meyer-Olkin (KMO) index, which assesses the appropriateness of applying Exploratory Factor Analysis (EFA) to the dataset of this study, yielded a KMO value of 0.916, indicating that the data were eligible for factor analysis. The Bartlett’s sphericity test yielded a significant result (χ 2 = 678.34, p-value < 0.001), indicating that the exploratory factor analysis (EFA) may be performed.

This study recruited participants from a heterogeneous community of university students. The recruitment procedure entailed distributing invites via university email lists, university-affiliated social media sites, and physical fliers displayed throughout the campus. Students who expressed interest were instructed to complete an online pre-screening questionnaire to confirm that they fulfilled the requirements for participation. These requirements included being currently enrolled in an undergraduate or graduate program, being at least 18 years old, and having a satisfactory level of proficiency in the language of instruction to comprehend and respond to the study materials. The exclusion criteria were established to eliminate part-time students, individuals under the age of 18, and non-students, in order to ensure that the research population was uniform in terms of their involvement with the university setting.

A grand total of 550 paper-and-pencil questionnaires were handed out to participants, of which 528 were successfully collected, yielding a response rate of 96%. Due to missing information, ten participants were omitted from the analysis, resulting in a final sample size of 518 responses.

## Results

### Descriptive statistics

Among the collected responses, 55.5% were categorised as male, while 45.5% were categorised as female. The distribution of students within the study sample was as follows: The sample consisted of students, and their distribution was as follows: 62.89% of the students were classed as undergraduate students and 37.11% were designated as postgraduate students. The participants’ age distribution was as follows: The age group of 18–25 years made up 33.26% of the sample, while the age group of 26–35 years accounted for 36.75% of the participants. The age group of 36–45 years formed 23.67% of the respondents, while individuals aged 45 and above constituted 6.32% of the population. The allocation of academic specialties in the institution is as follows: the School of Public Administration accounts for 24.56%, the School of Management accounts for 23.76%, the School of Economics accounts for 22.35%, and the School of Finance accounts for 29.33%.

### Validity, reliability, and multicollinearity analysis

Fornell and Larcker (1981) provide a set of prerequisites that must be met in order to evaluate the validity and reliability of a survey through structural equation modelling (SEM) analysis. For a latent variable to be considered legitimate, it must have a Cronbach’s alpha coefficient of 0.7 or higher. Table 2 shows that the Cronbach’s alpha values for each latent variable fulfil the set criteria, indicating that this study is genuine. Furthermore, Average Variance Extracted (AVE) is a commonly acknowledged metric for assessing reliability. Segars [67] suggested that in order to obtain approval in terms of reliability, the value of this index should be more than 0.5. This indication effectively conforms to the specified concepts and criteria. Therefore, the study model’s reliability is confirmed. Before evaluating the structural model, it is crucial to confirm that there is no linear relationship between the individual components. Hair, Black [68] deemed Variation Inflation Factor (VIF) values below 5 to be acceptable.

**Table 2:** Validity, reliability, and multicollinearity analysis

Variaibles	Cronbach Alpha	AVE	VIF
**Postgraduate**			
Emotional Intelligence	0.723	0.576	[3.67, 4.09]
Self-Efficacy	0.811	0.603	[3.13, 4.38]
Motivation	0.702	0.553	[2.98, 3.78]
Resilience	0.745	0.502	[3.11, 4.56]
Psychological Well-being	0.782	0.623	[2.54, 3.09]
**Undregraduate**			
Emotional Intelligence	0.764	0.555	[3.27, 4.19]
Self-Efficacy	0.787	0.529	[3.67, 4.05]
Motivation	0.811	0.588	[4.02, 4.77]
Resilience	0.823	0.623	[3.35, 4.72]
Psychological Well-being	0.709	0.689	[2.98, 3.97]

### Model fitting

**Fig. 2:**
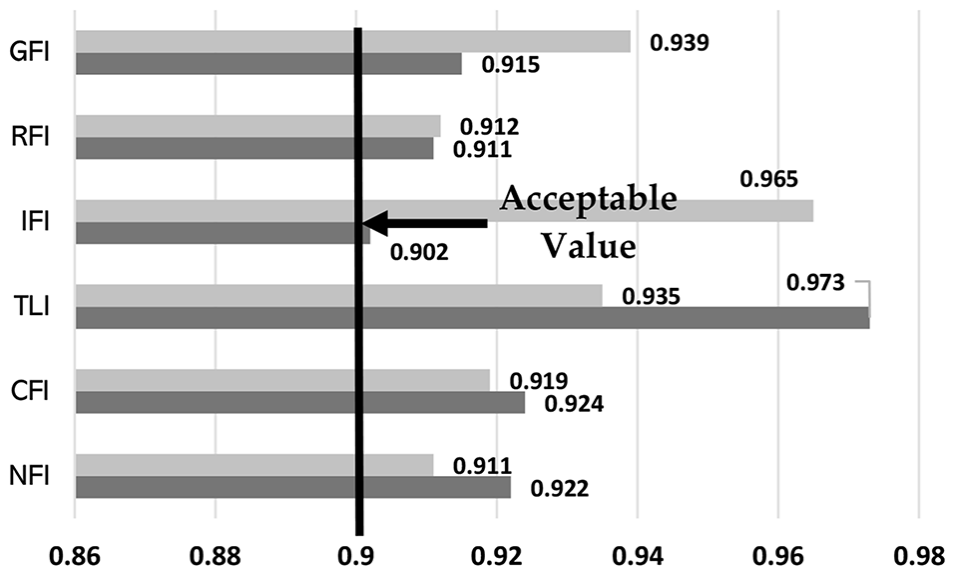
Fitting test

As per the findings of Hair, Ringle [69], it is advisable for a research model to get fit values that above 0.9. The study’s results demonstrate that the goodness of fit index (GFI), relative fit index (RFI), incremental fit index (IFI), comparative fit index (CFI), Tucker-Lewis index (TLI), and normed fit index (NFI) values were within acceptable ranges (refer to Fig. 2). In this study, the Chi-square/df value was discovered to be 1.116 In general, a Chi-square/df value of less than 2 suggests a strong model-data fit. Furthermore, we discovered that the RMSEA value was 0.049. A value of RMSEA less than or equal to 0.08 suggests an acceptable fit, whereas values less than 0.05 indicate a good fit. Thus, an RMSEA score of 0.049 indicates that the model fits the data well, with only a minor difference between the observed and predicted covariance matrices. Therefore, the level of agreement between the model and the data in this investigation was considered satisfactory.

### Common method variance

Common Method Variance, or CMV, is the difference between two sets of data that is caused by the way the data was measured instead of the things that the measurements are trying to measure. In behavioural research, this could be a problem, especially when one method (like a survey) is used to measure different constructs. To reduce the variation, we collected data anonymously and scored some things backwards. Once the data was gathered, Harman’s univariate factor analysis was used to see if there were any common-method variance. There were 21 factors with eigenvalues greater than 1. The first factor described 17.5% of the variation, which was less than the 40.0% critical standard [70]. It’s clear from this that the data in this study don’t have any major common-method variance.

### Structural model

The outcomes of structural equation modelling for both undergraduate and postgraduate students are presented in Tables 3 and 4. Emotional intelligence has been found to have a substantial positive link with self-efficacy, motivation, and resilience among both undergraduate and postgraduate students. There is a considerable correlation between self-efficacy and both academic accomplishment and psychological well-being. Motivation, as the second positive psychological characteristic, exhibited a significant positive correlation with both academic achievement and psychological well-being. Resilience is strongly correlated with both academic achievement and psychological well-being.

The significance of the partially mediated model was assessed using the bootstrap estimation process, with a selected bootstrap sample size of 5,000. The mediating effects of positive psychological characteristics are demonstrated in Tables 3 and 4. Table 3 illustrates the role of emotional intelligence in influencing the academic achievement of postgraduate and undergraduate students. Table 4 illustrates the role of emotional intelligence in influencing psychological well-being among postgraduate and undergraduate students.

In addition to the direct effects shown in Table 3, emotional intelligence had positive indirect effects on psychological well-being through self-efficacy, motivation, and resilience. Among undergraduate students, these effects were 0.184, 0.108, and 0.091 respectively. Among postgraduate students, the effects were 0.198, 0.180, and 0.117 respectively. Consequently, a positive correlation was found between increased emotional intelligence and elevated levels of self-efficacy, motivation, and resilience. These factors, in turn, were linked to enhanced psychological well-being among both postgraduate and undergraduate students. The indirect impacts of emotional intelligence on academic achievement through self-efficacy, motivation, and resilience were found to be 0.186, 0.110, and 0.093, respectively, for undergraduate students. For postgraduate students, the corresponding values were 0.199, 0.167, and 0.195. Consequently, a positive correlation was observed between increased emotional intelligence and elevated levels of self-efficacy, motivation, and resilience. These factors, in turn, were found to be linked to improved academic achievement in both undergraduate and postgraduate students.

**Table 3:** Direct, indirect, and total effects (Dependent Variable: academic achievement)

Relationships	Estimates	LL 95% CI	UL 95% CI
**Undergraduate Students (** ***N*** ** = 323)**			
*Direct Effect*			
Emotional Intelligence→ Self-efficacy	0.417***	0.316	0.486
Emotional Intelligence→ Motivation	0.368***	0.299	0.403
Emotional Intelligence→ Resilience	0.296**	0.211	0.342
Self-efficacy→ Academic Achievement	0.448***	0.407	0.486
Motivation→ Academic Achievement	0.299*	0.221	0.326
Resilience→ Academic Achievement	0.315**	0.259	0.371
Emotional Intelligence→ Academic Achievement	0.330***	0.278	0.376
*Indirect Effect*			
Emotional Intelligence→ Self-efficacy→ Academic Achievement	0.186*	0.102	0.201
Emotional Intelligence→ Motivation→ Academic Achievement	0.110*	0.078	0.123
Emotional Intelligence→ Resilience→ Academic Achievement	0.093*	0.063	0.108
Total Effect			
**Postgraduate Students (** ***N*** ** = 189)**			
*Direct Effect*			
Emotional Intelligence→ Self-efficacy	0.467***	0.408	0.522
Emotional Intelligence→ Motivation	0.399***	0.318	0.446
Emotional Intelligence→ Resilience	0.346***	0.321	0.388
Self-efficacy→ Academic Achievement	0.459***	0.386	0.463
Motivation→ Academic Achievement	0.421***	0.369	0.473
Resilience→ Academic Achievement	0.566***	0.511	0.616
Emotional Intelligence→ Academic Achievement	0.387***	0.319	0.427
*Indirect Effect*			
Emotional Intelligence→ Self-efficacy→ Academic Achievement	0.214**	0.169	0.227
Emotional Intelligence→ Motivation→ Academic Achievement	0.167*	0.119	0.206
Emotional Intelligence→ Resilience→ Academic Achievement	0.195*	0.139	0.267
Total Effect			

*<0.05; **<0.01; ***<0.001

**Table 4:** Direct, indirect, and total effects (Dependent Variable: Psychological Well-being)

Relationships	Estimates	LL 95% CI	UL 95% CI
**Undergraduate Students (** ***N*** ** = 323)**			
*Direct Effect*			
Emotional Intelligence→ Self-efficacy	0.414***	0.367	0.465
Emotional Intelligence→ Motivation	0.365***	0.317	0.429
Emotional Intelligence→ Resilience	0.293**	0.227	0.333
Self-efficacy→ Psychological Well-being	0.445***	0.406	0.486
Motivation→ Psychological Well-being	0.296**	0.239	0.367
Resilience→ Psychological Well-being	0.312**	0.267	0.365
Emotional Intelligence→ Psychological Well-being	0.327**	0.268	0.383
*Indirect Effect*			
Emotional Intelligence→ Self-efficacy→ Psychological Well-being	0.184*	0.123	0.217
Emotional Intelligence→ Motivation→ Psychological Well-being	0.108*	0.067	0.137
Emotional Intelligence→ Resilience→ Psychological Well-being	0.091*	0.067	0.119
Total Effect			
**Postgraduate Students (** ***N*** ** = 189)**			
*Direct Effect*			
Emotional Intelligence→ Self-efficacy	0.499***	0.437	0.522
Emotional Intelligence→ Motivation	0.396***	0.362	0.437
Emotional Intelligence→ Resilience	0.343***	0.319	0.386
Self-efficacy→ Psychological Well-being	0.497***	0.336	0.436
Motivation→ Psychological Well-being	0.455***	0.409	0.499
Resilience→ Psychological Well-being	0.344***	0.317	0.377
Emotional Intelligence→ Psychological Well-being	0.349***	0.307	0.393
*Indirect Effect*			
Emotional Intelligence→ Self-efficacy→ Psychological Well-being	0.248**	0.176	0.278
Emotional Intelligence→ Motivation→ Psychological Well-being	0.180*	0.113	0.203
Emotional Intelligence→ Resilience→ Psychological Well-being	0.117*	0.083	0.135
Total Effect			

*<0.05; **<0.01; ***<0.001

### Multigroup analysis

The study employed a thorough two-step process to thoroughly examine whether the associations between emotional intelligence, self-efficacy, motivation, resilience, psychological well-being, and academic achievement varied between two distinct groups: undergraduate and postgraduate students. This analysis, known as multigroup analysis, aimed to rigorously evaluate these relationships. Implementing the framework suggested by Angelini and Gini [71] for this section of the research implies using a systematic method that thoroughly examines the complex relationships between emotional intelligence, self-efficacy, motivation, resilience, psychological well-being, and academic achievement at different educational levels. At first, the analysis placed restrictions on each path coefficient for both groups, assuming that the influence of emotional intelligence on different outcomes such as self-efficacy, motivation, resilience, and their subsequent effects on well-being and academic success were the same regardless of educational level. Establishing a baseline comparison model is a vital step that assumes all associations are equal across groups, allowing for a rigorous examination of discrepancies.

After implementing these limitations, the study utilized the $\:Wald\:{\chi\:}^{2}$ test to thoroughly evaluate the accuracy of this assumption. The Wald χ^2^ test is a statistical method used to assess the significance of differences between models particular to different groups, while taking into account certain constraints. This test assesses whether the null hypothesis, which states that the path coefficients are the same for all groups, can be upheld or should be dismissed. If the $\:Wald\:{\chi\:}^{2}$ test yields a significant result, it would suggest that there are significant differences in the path relationships between undergraduates and postgraduates. This indicates that the educational context may influence the interaction between emotional intelligence, related psychological factors, and their impact on outcomes. In contrast, a result that is not statistically significant provides evidence in favor of the initial premise that there is no difference between the groups. This suggests that the ways in which emotional intelligence affects academic and well-being results are consistent across these educational levels.

The initial multigroup analysis using the $\:Wald\:{\chi\:}^{2}$ test revealed substantial group differences, as seen by the statistical result $\:{\chi\:}^{2}$= 65.33, with a 𝑝-value of 0.005. This substantial outcome resulted in the rejection of the null hypothesis that the route coefficients are similar between the undergraduate and postgraduate groups. This discovery implies that there are differences in the way emotional intelligence, self-efficacy, motivation, resilience, psychological well-being, and academic accomplishment are connected between these two educational levels. The findings shown in Table 5 demonstrate that the $\:Wald\:{\chi\:}^{2}$ test did not identify any noteworthy disparities in the indirect effects between the undergraduate and postgraduate student groups. This observation holds significance in the context of the study. More specifically, although there may be differences in the direct influence of certain factors, such as emotional intelligence, self-efficacy, motivation, resilience, psychological well-being, and academic achievement, the indirect effects of these factors on mediated relationships seem to operate similarly in both educational settings.

**Table 5:** Wald χ2 test for indirect effect

Relationships	$\:\varvec{W}\varvec{a}\varvec{l}\varvec{d}\:{\varvec{\chi\:}}^{2}$test	*P*-value
Emotional Intelligence→ Self-efficacy→ Academic Achievement	1.27	0.408
Emotional Intelligence→ Motivation→ Academic Achievement	1.38	0.321
Emotional Intelligence→ Resilience→ Academic Achievement	2.76	0.095
Emotional Intelligence→ Self-efficacy→ Psychological Well-being	2.11	0.123
Emotional Intelligence→ Motivation→ Psychological Well-being	1.93	0.243
Emotional Intelligence→ Resilience→ Psychological Well-being	0.97	0.567

## Discussion

The primary aim of this research is to examine the ways in which emotional intelligence contributes to the academic achievement and psychological well-being of students. The study incorporated positive psychology theory and emotional intelligence theory for the theoretical foundation. Emotional intelligence has an impact on an individual’s self-efficacy, resilience, motivation and at the same time on psychological well-being and academic achievement within the framework of emotional intelligence theory. The positive psychology paradigm mediating integrates motivation, self-efficacy, and self-efficacy in the relationship between emotional intelligence with both psychology well-being and academic achievement. The study also aimed to analyse the effects of emotional intelligence on self-efficacy, motivation, and resilience among Chinese students. The study also focused on the chain-mediating effects of self-efficacy, motivation, and resilience in the relationships of emotional intelligence with academic achievement and psychological well-being. There are several different ways in which the study makes new improvements to theory and practice.

First, the study adds to the body of research on emotional intelligence by discovering the strong positive links between emotional intelligence and positive psychological characteristics in Chinese students at both undergraduate and postgraduate levels. The implication is that emotional intelligence leads to positive psychological characteristics in individuals. The emotional intelligence changes also brought up self-efficacy, resilience, and motivation for both undergraduate and postgraduate Chinese students. The impact of emotional intelligence on self-efficacy [72, 73], resilience [74, 75] and motivation [29, 76] supported by previous studies in this area. The study demonstrates a high correlation between emotional intelligence and self-efficacy, which refers to an individual’s confidence in their capacity to accomplish objectives. One possible explanation for this finding is that students with high emotional intelligence possess a superior comprehension of their emotions and the impact these emotions can have on their performance. This level of consciousness enables individuals to tackle difficult activities with an optimistic attitude and a strong conviction in their own capabilities. They usually excel in receiving feedback, whether it is good or negative, and utilise it effectively to improve their abilities and understanding. In addition, students who possess a high level of emotional intelligence tend to be driven by internal factors, finding fulfilment in the process of learning and their own personal growth. According to Linnenbrink-Garcia, Patall [77], emotional awareness facilitates the alignment of academic attempts with personal beliefs and interests, hence enhancing the engagement and fulfilment in the learning process. In addition, they possess the skill to effectively handle emotions that could impede motivation, such as nervousness or displeasure. In addition, students with high emotional intelligence may effectively utilise positive emotions such as passion and curiosity, which in turn enhances their motivation [78]. Previous studies by Conradty and Bogner [79] and Murayama, FitzGibbon [80] found that intrinsic motivation plays a crucial role in achieving long-term academic achievement, as it is more enduring and self-rewarding in comparison to extrinsic drive. Another psychological characteristic that is improved by emotional intelligence is resilience, or the ability to bounce back quickly from problems. University life is often stressful, and students have to deal with academic stresses, social problems, and personal setbacks. Jordan and Troth [81] and Zhoc, King [82] thought that people with higher emotional intelligence are better able to deal with stress and problems. They usually have better ways of dealing with stress, like getting help, seeing things in a more positive light, and keeping a happy attitude. This mental flexibility helps them get back on their feet faster after a setback. Being able to relate to others makes their relationships with peers and teachers stronger, which is important for resilience because it gives them a support network.

Second, our study supports the existing notion in emotional intelligence theory that emotional intelligence has a major impact on both psychological well-being and academic accomplishment. This finding aligns with previous research in the field. Prior studies have typically investigated emotional intelligence and its effects on psychological well-being [12, 44, 73] and academic accomplishment [2, 83] as separate entities, which has restricted the comprehension of how these factors may interact within a unified and comprehensive framework. Our study makes a distinct contribution to the area by incorporating psychological well-being and academic accomplishment as target variables in the same model. This approach offers a comprehensive perspective on how emotional intelligence simultaneously affects two important parts of university students’ lives.

Through the utilization of emotional intelligence framework, encompassing the capacities to notice, use, comprehend, and regulate emotions, we have observed that students possessing elevated levels of emotional intelligence are more adept at managing the pressures and requirements of university life. These pupils exhibited improved psychological well-being through proficiently controlling and overseeing their emotions, resulting in decreased stress levels and greater general mental health. In addition, their academic performance was enhanced by their increased emotional awareness and ability to regulate their emotions, resulting in improved concentration, enhanced study habits, and more effective interactions with classmates and teachers. These results validate the crucial significance of emotional intelligence in creating a conducive atmosphere for students to attain academic excellence while also preserving their mental well-being, hence demonstrating the tangible implementation of emotional intelligence theory in educational contexts.

Third, the study adds valuable results to the positive psychological theory literature in educational and health science studies. The study revealed a significant positive relationship between positive psychological characteristics and both psychology well-being and academic achievement. Previous studies have found that positive psychological characteristics have large effects on both academic achievement [84–86] and psychology well-being [87, 88]. However, there is a lack of study looked at both factors together. But it’s important to look at both academic success and student well-being together to get a full picture of school success. In the past, academic success was the main goal of schools, and grades, test scores, and finish rates were often used to measure this. But this narrow focus can mean that the bigger picture of a student’s life and health is missed, even though these are important for long-term and worthwhile success.

Forth, this study presents a novel perspective by introducing positive psychological characteristics as a mediator to examine the association between emotional intelligence and both psychological well-being and academic accomplishment. The study found that emotional intelligence is linked to psychological well-being and academic achievement through the mediation of positive psychological characteristics such as self-efficacy, motivation, and resilience. We introduced a sophisticated framework, as seen in Fig. 1. Prior research has primarily focused on examining one or two positive psychological characteristics as potential mediators among human behaviour studies [89, 90]. A study conducted by Chang and Tsai [28] examined the relationship between emotional intelligence, resilience, self-efficacy, and academic accomplishment in online classrooms.

Fifth, our study investigated how self-efficacy, motivation, and resilience mediate the relationship between emotional intelligence and outcomes such as psychological well-being and academic achievement. We found that there is a notable difference in these relationships between undergraduate and postgraduate students. The contrast mostly arises from the intricate and rigorous nature of postgraduate education, which typically necessitates a higher level of self-regulation, sophisticated problem-solving abilities, and superior emotional control. Postgraduate students, who generally have a greater accumulation of life and academic experiences, has heightened emotional intelligence that greatly strengthens their self-confidence. This heightened self-efficacy not only boosts their confidence in addressing demanding academic tasks [27, 91] and intricate research projects but also amplifies their overall motivation [36, 92]. This motivation is essential, as it drives individuals through the challenging requirements of postgraduate studies and maintains them at periods of possible academic exhaustion or disappointments.

Last, our study indicates that although the immediate impacts of variables such as emotional intelligence, self-efficacy, motivation, resilience, psychological well-being, and academic achievement may vary between undergraduate and postgraduate students, the indirect relationships between these factors remain consistent across both educational levels. In essence, although students at various academic levels may experience these effects in different ways on a surface level, the fundamental mechanisms through which these factors interact indirectly (such as how emotional intelligence impacts academic achievement through resilience or motivation) appear to function similarly regardless of whether a student is an undergraduate or a postgraduate [93, 94]. This discovery emphasizes the inherent essence of these relationships and indicates that approaches focused on improving these characteristics could be advantageous in various educational settings.

### Practical implications

The effects of emotional intelligence on the psychological well-being and academic achievement of university students, mediated by positive behavioural characteristics, have significant practical implications for educators, university officials, and students themselves. Gaining a comprehensive understanding of these consequences can result in the development of more efficient approaches to improve student achievement and general well-being. Here are several significant practical consequences:

- Integrating emotional intelligence and positive psychology approaches into the curriculum can equip students with valuable competencies. Enrolling in courses or seminars that specifically target self-awareness, empathy, stress management, and resilience might assist students in effectively navigating the difficulties associated with university life. In addition, implementing instructional approaches that promote cooperation, communication, and reflection can create a learning atmosphere that is favourable for the cultivation of emotional intelligence.
- Universities ought to allocate resources towards the establishment of counselling and mental health services that explicitly target the cultivation of emotional intelligence and positive psychological characteristics. Offering resources and assistance for stress management, coping mechanisms, and emotional control can greatly enhance the psychological well-being and academic achievement of students.
- Providing training to faculty members to identify and assist with the emotional and psychological needs of students might yield significant advantages. Faculty members can acquire the ability to recognise indications of emotional distress, offer compassionate assistance, and direct students to suitable resources.
- By incorporating emotional intelligence and positive psychology principles into career counselling, students can enhance their ability to make well-informed and emotionally intelligent choices regarding their careers. By comprehending their aptitudes, principles, and emotional skills, individuals can effectively select career trajectories that are in harmony with their personal and professional objectives.

### Limitations and prospects for future research

- Cross-sectional studies yield data collected at a certain moment in time. Due to the absence of data regarding the evolution of variables over time, cross-sectional studies are unable to depict the progression of development or the potential impact of individual changes in emotional intelligence on academic achievement or psychological well-being throughout a student’s university tenure. In order to address this limitation, it is advisable to employ a longitudinal study design. Longitudinal studies enable researchers to study the development and fluctuations of emotional intelligence and positive psychological characteristics in university students over time. These studies also investigate the relationship between these changes and variances in academic achievement and psychological well-being. Self-reported surveys were used extensively in this study.
- Self-reported measures are always going to be subjective. This tendency for people to show themselves in a better light, whether they are aware of it or not, is called social desirability bias. This can cause people to over- or under-rate things like psychic health or emotional intelligence. Also, self-reported data rely on the person being able to remember and report feelings, behaviours, or events correctly. This recall might not be perfect, which could mean that the info is wrong. Use qualitative methods like focus groups and conversations. Some of these can give you more information about what students have been through and how they see things than a questionnaire could have.
- A potential limitation of our study on the mediation effects of self-efficacy, motivation, and resilience between emotional intelligence and outcomes such as psychological well-being and academic accomplishment is the presence of unaccounted mediators and moderators. Although our attention was primarily directed towards these particular psychological characteristics, it is important to acknowledge that there may be other factors that could potentially impact these associations. Personality factors like conscientiousness and openness, social support systems, and previous academic experiences can also have important effects as mediators or moderators. These elements have the ability to modify the way emotional intelligence affects academic and psychological outcomes.
- A major limitation of our study is that it only included Chinese university students as participants. The narrow demographic scope of our study limits the applicability of our results to university students in other nations or to persons belonging to different age cohorts. The cultural, educational, and developmental environments have a substantial impact on psychological dimensions such as emotional intelligence, motivation, resilience, and how they affect psychological well-being and academic accomplishment. For example, variations in emotional expression and regulation due to cultural differences might impact the way emotional intelligence is demonstrated and functions in various environments. Similarly, the global variation in educational systems and their inherent demands can potentially affect the influence of motivation and resilience on academic achievements.

## Data Availability

No datasets were generated or analysed during the current study.

## Abbreviations

EI: Emotional intelligence
SEA: Self-emotion appraisals
OEA: Others’ emotion appraisals
ROE: Regulation of emotion
UOE: Use of emotion
SPWB: Scales of Psychological Well-being
GPA: Grade point average
SEM: Structural equation modelling
AVE: Average Variance Extracted
VIF: Variation Inflation Factor
GIF: Goodness of fit index
RFI: Relative fit index
IFI: Incremental fit index
CFI: Comparative fit index
TLI: Tucker-Lewis index
NFI: Normed fit index
CMV: Common Method Variance
